# Yale insulin protocol infusion in sepsis patients

**DOI:** 10.1186/cc10374

**Published:** 2011-10-27

**Authors:** S Oliveira

**Affiliations:** 1 Albert Schweitzer and Hospital Bangu Hospital, Rio de Janeiro - RJ, Brazil

## Introduction

Tight glycemic control is a major concern in critical care. The objective of this study in the ICU was to evaluate the effectiveness and safety of the Yale insulin infusion protocol in sepsis patients.

## Methods

A retrospective, before-after cohort study. Selected end-points were mean blood glucose levels, time to reach the target range of 100 to 150 mg/dl, percentage of blood glucose in the target range, and hypoglycemia incidence.

## Results

Were studied 78 patients: 42 in the control group (CG) and 36 in the protocol group (PG). Bedside blood glucose was measured 3,755 times for a mean value of 134.1 ± 15.4 mg/dl in the PG versus 1,730 times for a mean value of 172.7 ± 33.6 mg/dl in the CG. Blood glucose values were in the target range 63% and 37% of the times, respectively, for the PG and the CG (*P *< 0.001). The median time to reach the glucose target range was 8 hours (range 5 to 17 hours) for the PG and 53 hours (range 23 to 218 hours) for the CG (*P *< 0.001). The incidence of severe hypoglycemia reached a statistically significant difference: one patient in the PG versus four patients in the CG (*P *< 0.01). All patients reached the target in 72 hours of insulin infusion in the PG while only 29 patients in the CG reached this target.

## Conclusion

The Yale insulin infusion protocol was effective and safe in sepsis patients admitted to the ICU.

